# Gratitude and Social Media: A Pilot Experiment on the Benefits of Exposure to Others’ Grateful Interactions on Facebook

**DOI:** 10.3389/fpsyg.2021.667052

**Published:** 2021-05-12

**Authors:** Simona Sciara, Daniela Villani, Anna Flavia Di Natale, Camillo Regalia

**Affiliations:** ^1^Department of Psychology, Catholic University of the Sacred Heart, Milan, Italy; ^2^UniSR-Social.Lab, Faculty of Psychology, Vita-Salute San Raffaele University, Milan, Italy; ^3^Faculty of Psychology, University of Milan-Bicocca, Milan, Italy

**Keywords:** gratitude, social media, well-being, social learning, emotional contagion, positive psychology, social networking sites, Facebook

## Abstract

Facebook and other social networking sites allow observation of others’ interactions that in normal, offline life would simply be *undetectable* (e.g., a two-voice conversation viewable on the Facebook wall, from the perspective of a real, silent witness). Drawing on this specific property, the theory of social learning, and the most direct implications of emotional contagion, our pilot experiment (*N* = 49) aimed to test whether the exposure to others’ grateful interactions on Facebook enhances (a) users’ felt gratitude, (b) expressed gratitude, and (c) their subjective well-being. For the threefold purpose, we created *ad hoc* Facebook groups in which the exposure to some accomplices’ exchange of grateful messages for 2 weeks was experimentally manipulated and users’ felt/expressed gratitude and well-being were consequently assessed. Results partially supported both hypotheses. Observing others’ exchange of grateful posts/comments on Facebook appeared to enhance participants’ in-person expression of gratitude (i.e., self-reported gratitude expression within face-to-face interactions), but not their direct and subjective experiences of gratitude. Similarly, exposure to others’ grateful messages improved some components of subjective well-being, such as satisfaction with life, but not negative and positive affect. Taken together, however, our preliminary findings suggest for the first time that social networking sites may actually amplify the spreading of gratitude and its benefits. Implications of our results for professionals and future research in the field of health, education, and social media communication are discussed.

## Introduction

In the past 10 years, the use of *social media* among young people has become a common practice in everyday life (Faelens et al., 2021). Social media such as Facebook, Twitter, and Instagram allow for the posting, reading, and sharing of audio, video, or textual contents in a fast and easy manner, turning into powerful communication channels. This phenomenon comes with relevant psychological implications. Up to now, results of studies investigating the associations between social network site (SNS) use and well-being are mixed (Huang, 2017; Verduyn et al., 2017); and empirical evidence suggests that how users are interacting with SNS platforms is critical for their well-being (Odgers and Jensen, 2020). In particular, passively browsing SNS represents a type of activity negatively associated with well-being (Thorisdottir et al., 2019), even if recent studies showed the importance of deepening this analysis by considering also *who* and *what* individuals are browsing (Burnell et al., 2020).

Only recently are researchers considering the peculiar relationship between social media and gratitude (Renshaw and Hindman, 2017; Koay et al., 2020). Gratitude has been defined as the life orientation toward noticing and appreciating the positive in the world that entails, among others, the tendency to experience grateful feelings (Wood et al., 2008). More technically, gratitude is a positive emotion that can be experienced and expressed when a person appraises that another person (i.e., the benefactor) did something notable—without expecting something in return—to intentionally benefit another person or the self (Ortony et al., 1988; Algoe et al., 2008; Algoe and Haidt, 2009). Benefits of gratitude have been broadly studied and documented in psychological literature. Feeling grateful has been demonstrated to enhance physical health (Emmons and McCullough, 2003), augment life satisfaction (Lambert et al., 2009), and improve both negative and positive affectivity (Renshaw and Rock, 2018). Accordingly, gratitude-based interventions, as well as gratitude expression in general, have been shown to improve people’s well-being, by augmenting overall subjective well-being, mental health (Emmons and McCullough, 2003; Froh et al., 2008; Killen and Macaskill, 2015), self-esteem (Rash et al., 2011), prosociality (Grant and Gino, 2010; Ma et al., 2017; Tsang and Martin, 2019), and the quality of close relationships (Lambert et al., 2010).

Recent studies demonstrated that benefits of gratitude-based interventions can be achieved even when they occur via instant communication technologies like Facebook and Instagram (Renshaw and Hindman, 2017; Koay et al., 2020). Renshaw and Hindman (2017), for example, adopted a gratitude intervention technique, namely, the letter-writing-and-visit gratitude intervention, using notes and instant communication. Specifically, they asked a group of participants to use instant messaging to express gratitude to someone three times a day for 2 weeks. Two control groups were instructed to either use, three times a day for 2 weeks, instant messaging or to access a private journal, to write about something they recently learnt in their college courses. Similarly, Koay et al. (2020) adapted the daily listing gratitude intervention to Instagram. In particular, they asked an experimental group to post images related to gratitude, such as a picture of something they were grateful for, on a daily basis over 7 days. Similarly, they asked a control group to post pictures referring to colors describing, for example, their feelings. In both the above-mentioned studies (Renshaw and Hindman, 2017; Koay et al., 2020), initial findings indicate that social media-based gratitude interventions have positive effects on gratitude and some indicators of subjective well-being.

While these are first attempts to investigate how social media may serve as tools to sustain gratitude-based interventions, to date, no research has ever focused on the *specific* role that social media may play in the diffusion of gratitude and its benefits. Doubtless, social networking sites cannot be seen as mere channels to communicate gratitude to others. A very interesting and—surprisingly—under-explored property of social media is that they allow users also to observe other users’ two-way interactions (e.g., Rau et al., 2008). Sites like Facebook and Instagram, in fact, have *unique* features that give people possibilities they have never had before and that may potentially affect also gratitude diffusion (e.g., Rau et al., 2008; see Kaplan and Haenlein, 2010, for a description of the main features of social networking sites). On a social networking site like Facebook, it is quite common to witness posted conversations in which two or more users chat about things they usually discuss in person, without a public, or that clearly address only some users and not all the people from whom these posts can be viewed. Posting congratulations on the recent success of a friend, declaring love to someone, the organization of a forthcoming small meeting, and the exchange of jokes about a new TV program are all good examples. In all these cases, in fact, other users observe public interactions that in normal, offline life would simply be *undetectable* (i.e., two-voice conversations that are normally private but that, in these cases, are viewable on the Facebook wall, as from the perspective of a real silent witness).

The fact that social media allow the observation of other users’ conversations could be relevant in the diffusion of gratitude and its benefits in two main respects. First, observing others is a primary way to learn grateful behaviors. In this sense, social media would represent perfect environments in which users may observe others and imitate their behaviors without efforts or active intentions (Non-necke et al., 2004; Preece et al., 2004; Rau et al., 2008) and, even spontaneously, as passive observation of others is frequently recognized as a drive for using social media by users themselves (e.g., Tosun, 2012). Second, as emotions expressed by others through social media posts may influence individuals’ own emotions (Kramer et al., 2014), observing others’ grateful expressions on social sites could potentially have an impact on users’ own gratitude, resulting in augmented gratitude feelings and respective benefits on well-being. In other words, the opportunity to passively observe other users’ behavior and learn from others’ grateful interactions makes social networking sites a powerful tool for amplifying the spreading of gratitude and, especially, the diffusion of its positive effects on well-being.

### Learn and Benefit From Observing Others’ Grateful Interactions on Facebook

According to the theory of social learning (Bandura, 1968), observing other’s interactions could make people apprehend behaviors of any sort, including grateful behaviors. As originally described by the theory, observational learning is the ability to learn new responses as a result of observing behavioral models (Bandura, 1977, 1968). Importantly, as observation and imitation are spontaneous phenomena, simple exposure to others’ attitudes and behaviors can cause the natural acquisition of such attitudes and behaviors, also when learning is not planned (Bandura, 1971; see also Browder et al., 1986). Through observation, people can indeed learn attitudes, feelings’ expressions, and specific emotional behaviors (Kanekar, 1973, 1976). Children, for instance, learn emotional expressions by observing how others express and describe feelings, but also by seeing the consequences that occur because of such displays (Kramer, 2014). With respect to the specific case of gratitude, some correlational studies clearly suggest that also children’s gratitude could be the result of observation and imitation of parents (Greif and Gleason, 1980; Hoy et al., 2013; Rothenberg et al., 2017; Hussong et al., 2019). Thus, it is reasonable to expect that users exposed to grateful behaviors on social media will be likely to imitate such behaviors, showing a more frequent expression of gratitude.

Despite that no research has ever explicitly investigated effects of observational learning within social media contexts, evidence for learning as a result of social media usage does already exist. In recent years, social media have been presented as a suitable tool for learning (e.g., Hong et al., 2016). Social networking sites, and Facebook more than others, are places in which users can communicate and exchange information in an immediate and easy way, learning from each other through reciprocal sharing and comments, as well as through exposure to both entertainment and informative contents (see Manca and Ranieri, 2013 for a review). Van Puijenbroek et al. (2014), for instance, demonstrated that social media use can effectively support work-related learning among employees. Likewise, Mbati (2013) showed that online discussion forums are ideal for the stimulation of constructivism and observational learning in online learning programs. Always from the field of education, many studies reported that social media can create learning environments that support student learning (Dabbagh and Kitsantas, 2012). Taken together, all these findings suggest that social sites can potentially improve learning with respect to any learning object, including the case of gratitude.

Another phenomenon that is relevant for understanding the role of social media in gratitude diffusion is emotional contagion (Levy and Nail, 1993; Hatfield et al., 1994), i.e., the well-known type of social influence in which people’s emotions and behaviors are influenced by the mere observation of others’ emotional expressions (e.g., Kelly and Barsade, 2001). Interestingly, a recent research by Algoe et al. (2020) demonstrated through eight experiments that observing a gratitude social interaction is sufficient to make a witness to that interaction more helpful and affiliative toward both the actors of the interaction (i.e., the so-called “witnessing effect”; see Algoe et al., 2020). To our best knowledge, Algoe and colleagues’ finding is the very first evidence of how social consequences of gratitude could work at a *group* level—an evidence that could help in clarifying how gratitude spreads and how such spreading could be encouraged within modern societies. In this sense, the role of social media seems pivotal.

As already shown by several studies, emotional contagion is a widespread effect on social media sites and other computer-mediated communications (Cheshin et al., 2011; Coviello et al., 2014; Ferrara and Yang, 2015; Xiong et al., 2018). Many results indicate, for instance, that emotions expressed by others on Facebook actually influence users’ own emotions, demonstrating that in-person interaction and non-verbal cues are not necessary for emotional contagion to occur (Kramer et al., 2014; see also Del Vicario et al., 2016). Moreover, observation of others’ positive experiences on Facebook constitutes itself a positive experience for users (Kramer et al., 2014), suggesting that benefits of positive emotions could be experienced by users even when they are simply observing. Therefore, it is possible that others’ expressed gratitude on Facebook could influence individual’s own feeling of gratitude, as well as the experiences of its numerous benefits. Thus, in line with the same reasoning, as gratitude improves subjective well-being and general mental health, observing others’ grateful interactions should be sufficient—at least to a minimum extent—to make people benefit from such expressions of gratitude and to experience (a) augmented feelings of gratitude and (b) an increased well-being.

In sum, as an inference of both observational learning and emotional contagion, learning gratitude through observation of others on Facebook, and also benefit from others’ manifestations of gratitude, really seem possible. Moreover, the exposure to others’ grateful behaviors on Facebook should make users not only imitate such behaviors (social learning effect) but also benefit from the positivity that such behaviors induce in persons who are expressing or receiving grateful messages (emotional contagion effect). In this sense, Facebook would represent a singular tool for spreading grateful attitudes and, especially, amplifying its benefits.

### The Present Research

The present pilot study is intended to be an innovative small-scale applied research to analyze the effects of exposure to others’ grateful interaction by using a social media, Facebook, as an ecological setting. Specifically, we applied the theory of social learning (Bandura, 1968, 1977) and the most direct implications of emotional contagion (Hatfield et al., 1994) to hypothesize specific effects of exposure to others’ grateful interactions via Facebook on felt gratitude, expressed gratitude, and well-being. Precisely, we intended to test *three* main hypotheses. The first hypothesis was that exposure to some accomplices’ grateful interactions on Facebook would cause an augmented tendency to experience gratitude, both in terms of one’s own feelings and in terms of awareness of others’ feelings. The second hypothesis was that such an exposure to grateful interactions would cause more frequent expression of gratitude. Finally, the third hypothesis stated that exposure to the same accomplices’ grateful behaviors would also ameliorate Facebook users’ well-being; in particular, we expected that observing others’ gratitude exchange would improve two components of subjective well-being, i.e., satisfaction with life and positive affect. For the threefold purpose, we created *ad hoc* Facebook groups in which exposure to accomplices’ exchange of grateful messages was experimentally manipulated.

## Materials and Methods

### Participants and Design

Forty-nine Italian young adults (46.90% females; mean age = 26.63 years, *SD* = 4.38) volunteered in a 2 (*Gratitude exposure on Facebook*: control vs. gratitude group) × 2 (*Time*: pre- vs. post-manipulation) repeated-measures experiment, in which *felt gratitude*, *expressed gratitude*, and *well-being measures* served as dependent variables. Felt and expressed gratitude were assessed once, right after the manipulation, whereas well-being measures were collected twice, both before and after the manipulation. At the onset of the study, we also measured participants’ *trait gratitude* in order to control for any effect of the most relevant dispositional factor. With this small sample, the study had 80% power to detect an effect size of at least *f*_(_*_U_*_)_ = 0.42 (i.e., a *medium-to-large* effect according to Cohen, 1988) in within-between interaction effects (*α* = 0.05; non-centrality parameter *λ* = 8.18; critical *F* = 4.05; numerator *df* = 1; denominator *df* = 47; see *G^∗^Power 3.1*, Faul et al., 2007). In this respect, it should be noted that we purposely maintained the sample size so small to encourage social interactions within the Facebook group, in order to avoid potential embarrassment due to speaking to a too large unknown audience.

### Procedure

At the beginning of the procedure, the study was introduced by email to participants as exploring people’s interests in psychosocial themes and especially aimed at investigating users’ everyday behavior on social media (*cover story*). Then, participants expressed their informed consent to participate in the study and filled out a first online questionnaire (*pre-manipulation questionnaire*). The questionnaire requested basic demographics, asked participants about their general habits and preferences on social media, and then introduced the pre-experimental measures—i.e., the measurement of trait gratitude and the first assessment of well-being.

To manipulate the exposure to others’ grateful interactions, participants were told to freely use a Facebook group for about 2 weeks. The instructions proposed were consistent with the pragmatic approach (Patsopoulos, 2011), aimed at supporting behaviors that the participants would naturally implement in ecological settings and to maximize the applicability and generalizability of the study. The Facebook group was intended to share some interesting notions from psychology and spread the latest discoveries from psychosocial research. Then, to implement the manipulation, right before the invitation to join the group, we randomly assigned participants to one of two experimental conditions—i.e., one of two *apparently identical* groups on Facebook. In the first Facebook group (*gratitude group*), participants were exposed to some accomplices’ grateful interactions, while in the second Facebook group (*control group*), participants were exposed to similar interactions but, this time, without references to gratitude (see the *Manipulation of Exposure to Others’ Grateful Interactions* subsection for details and some practical examples).

After the manipulation, participants filled out the second online questionnaire (*post-manipulation questionnaire*). This second questionnaire contained all the post-experimental measures—i.e., the felt gratitude scale, the expressed gratitude items, and the second assessment of well-being. Finally, participants were extensively debriefed and thanked for participation. The protocol was approved by the Ethical Committee of the Department of Psychology of the Catholic University of the Sacred Heart of Milan, Italy.

### Background Measures

#### Participants’ General Habits and Preferences on Social Media

Participants’ general habits and preferences on social media were assessed at the onset of the experiment with some basic questions, such as “*What’s your favorite social networking site?*” and “*How much frequently do you use Facebook groups?*” If pertinent, answers were provided on a unipolar Likert-type scale (1 = “*not at all*” to 7 = “*very much*”).

#### Participants’ Trait Gratitude

We assessed participants’ trait gratitude to be able to control, later in our analyses, for the most relevant dispositional differences that participants were likely to show. At the onset of the study, participants filled out the Italian version of the Gratitude Questionnaire (GQ-6) for the assessment of dispositional gratitude (six items; Cronbach’s *α* = 0.61; see McCullough et al., 2002; Caputo, 2016 for the original version).

### Manipulation of Exposure to Others’ Grateful Interactions

Before implementing the manipulation, we purposely created two almost identical closed groups on Facebook. Both groups were named “*Anche io psico*” (literally translated as “I psycho too”) and were intended to share notions from psychology and spread discoveries from psychosocial research. For the scope, two psychologists daily posted the same themed contents *in parallel*, on both groups (e.g., posts, links, and brief texts regarding psychological themes). Such contents were primarily intended to maintain the plausibility of the cover story and, additionally, to stimulate participants’ active use of the Facebook groups. Notably, none of these posts regarded gratitude.

To manipulate the exposure to others’ gratitude, participants were randomly assigned to one of two experimental conditions—i.e., one of the two *apparently identical* groups on Facebook. In both conditions, participants were told to freely and spontaneously use the group for 2 weeks (also sharing some off-topic posts if they wanted) and were all exposed to exactly the *same* contents the psychologists posted every day. The only exception regarded some bogus interactions daily acted by accomplices. In the first Facebook group (*gratitude group*), participants were exposed to accomplices’ grateful interactions—i.e., public, grateful interactions acted by accomplices through posts and comments. In the second Facebook group (*control group*), instead, participants were exposed to similar interactions but, this time, net of any reference to gratitude—i.e., the same (bogus) interactions without grateful words (see Algoe et al., 2020, for similar manipulations of exposure to others’ grateful interactions). Importantly, all participants were definitely unaware of the existence of two alternative Facebook groups. With respect to this, it is also important to note that we originally planned to exploit a unique Facebook group, in which we would simply manipulate *visibility* of accomplices’ posts; however, Facebook does not allow users to administer visibility of posts within its closed groups. For this reason, a clearer experiment in which all participants share the same virtual space while observing different contents was impossible to conduct.

**FIGURE 1:**
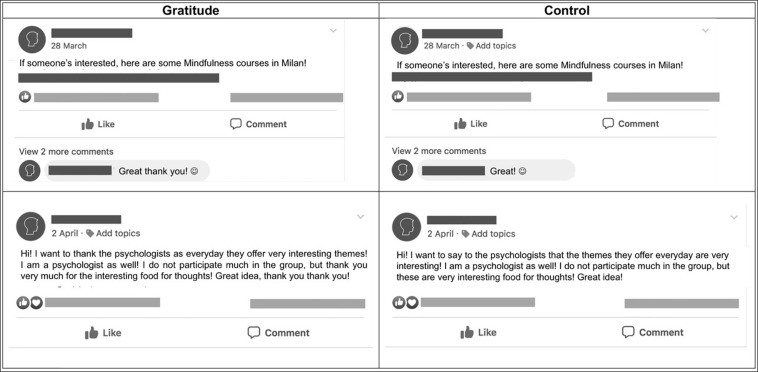
Some examples of Facebook posts used by accomplices to implement the manipulation of gratitude exposure: an action-and-response chat posted in the *gratitude group* **(top left)**; the same action-and-response chat posted in the *control group* **(top right)**; a single public post, addressed to all group’s members posted in the gratitude group **(bottom left)**; and the same single public post, posted in the control group **(bottom right)**. Posts’ original content was in Italian; the same content is here translated from Italian into English for example purposes.

Accomplices’ false interactions occurred once a day in both groups (with vs. without gratitude), for the entire duration of the Facebook group’s life. Precisely, a dozen accomplices (i.e., 12 different people) alternated two different forms of Facebook interaction; posts could be in the form of an action-and-response public chat (e.g., Accomplice A posts a brief text tagging Accomplice B; Accomplice B answers with a comment) or in the form of a single public post, addressed to all group’s members (e.g., Accomplice A posts a brief text with a photo). For example, in an action-and-response bogus interaction, Accomplice A posted a tip for anyone interested in starting the practice of mindfulness; Accomplice B commented below with the following response: “Thank you very much, it’s very interesting” (*gratitude group*) vs. “It’s very interesting” (*control group*). In another example, Accomplice A posted the following statement: “Hi guys! I’m very happy because I’m going to eat this delicious pasta. Thank you, mom! [Sharing a photograph of a meal]” (*gratitude group*) vs. “Hi guys! I’m very happy because I’m going to eat this delicious pasta! [Sharing the same photograph]” (*control group*). Some other examples of the manipulation’s bogus interactions are depicted in Figure 1.

Concerning our manipulation of gratitude exposure, it is important to highlight that the words “thank you” added in the Facebook posts and comments were the only difference between the gratitude and control conditions. Precisely, to create our two conditions, we carefully followed the lead of Algoe et al. (2020). Across several experiments, the authors manipulated gratitude exposure by varying comments previously left on a Word file that participants had to re-review: “In the gratitude expression condition, there was simply one extra comment […] saying ‘Thank you so much for catching those typos!’ The control condition did not include an extra comment” (p. 48, Algoe et al., 2020). Our procedure entailed similar little variations but repeated them daily.

Finally, the adding of the words “thank you” should not be seen as an oversimplification of gratitude. According to the definition of gratitude (Ortony et al., 1988; Algoe et al., 2008; Algoe and Haidt, 2009), in fact, the words “thank you” convey a unique, specific meaning since they reveal that the speaker has recognized another person’s free and positive action and—based on this recognition—has decided to let this person know of such recognition and respective positive emotion. For this reason, many gratitude-based interventions require people not to simply express their emotions to their benefactors but also to explicitly express gratitude, i.e., by saying “thank you” (e.g., Lambert et al., 2010). Without saying “thank you,” such expressions would have other meanings and bring different effects (see also Algoe et al., 2020).

### Dependent Measures

#### Measurement of Felt Gratitude

To measure feelings of gratitude after the manipulation, participants filled out an *ad hoc* questionnaire. Since we hypothesized that participants could feel more gratitude because of the exposure to others’ grateful interactions, we included not only items concerning pure, direct feelings of gratitude but also—on exploratory bases—two items tapping into the feeling of gratitude resulting from a resonance reaction to others’ gratitude. Such a distinction was also intended to enable us—later in our analysis—to potentially diversify among *direct* feelings of gratitude and *indirect* feelings of gratitude, i.e., feelings due to a resonance reaction induced by the noticing of others’ gratitude. Consistent conceptualizations can be found in the report of a series of studies on the effects of the appraisal of others’ positive actions on emotions like gratitude, described as an “other-praising emotion,” i.e., stemming from a focus on others and dependent on the noticing of others’ positive actions and emotions (Algoe and Haidt, 2009).

Based on the above rationale, our scale consisted of eight items assessing participants’ *feeling of gratitude* during the last 2 weeks, with six questions tapping into a direct experience of one’s own gratitude (e.g., “*In the last 2 weeks, did you feel grateful?*” and “*Did you feel grateful toward a person that you had never felt grateful to?*”) and two questions measuring the feelings of gratitude experienced as a resonance reaction due to the noticing of others’ gratitude (e.g., “*In the last 2 weeks, did you feel/sense others’ gratitude?*”). Answers were provided on unipolar Likert-type scales (1 = “never” to 5 = “very frequently”). The Cronbach alpha for the total scale was high (Cronbach’s α = 0.90; eight items), as well as for both the subscales (*α* = 0.88, six items, for the subscale measuring direct feelings of gratitude; *α* = 0.81, two items, for the subscale measuring the felt resonance of others’ feelings of gratitude).

#### Measurement of Expressed Gratitude

Four items assessed participants’ frequency of *expression of gratitude*, through four different channels—i.e., in person, via telephone calls, via instant messages (e.g., WhatsApp messages and Messenger messages), and via social media public posts (e.g., Facebook wall posts and Instagram feeds), always during the last 2 weeks (e.g., “*During the last 2 weeks, did you use instant messages to express gratitude?*”). Answers were provided on unipolar Likert-type scales (1 = “never” to 5 = “very frequently”). Notably, we asked participants about various channels only to be exhaustive in detecting their (self-reported) behaviors. In other words, we did not have precise hypotheses concerning the specific channels the participants would have used but only wanted to detect influences of gratitude exposure on expressed gratitude regarding any channel.

#### Measurements of Well-Being

Both before and after the manipulation, we assessed both dimensions of subjective well-being: life satisfaction and negative/positive affectivity. Participants filled out the Italian version of the Satisfaction With Life Scale (SWLS), for the evaluation of cognitive dimension of subjective well-being and global life satisfaction (five items; Diener et al., 1985; Di Fabio and Busoni, 2009; Di Fabio and Palazzeschi, 2012 for the original version), and, then, the Italian version of the Positive and Negative Affect Scale (PANAS), for the evaluation of affective dimension of subjective well-being during the last month (20 items; see Watson et al., 1988; Terracciano et al., 2003 for the original version). In the current study, the Cronbach alpha coefficients of measures of well-being were 0.79 for the SWLS, 0.87 for the Positive Affect subscale of PANAS, and 0.85 for the Negative Affect subscale of PANAS.

## Results

### Preliminary and Descriptive Analyses

Overall, participants’ trait gratitude, as measured at the onset of the experiment, was comparable among conditions. Indeed, there was no significant difference in the GQ-6 score between the control group (*M* = 32.32, *SD* = 4.50; bootstrap 95% *CI* with 5,000 resamples [30.43, 33.96]) and the gratitude group (*M* = 30.54, *SD* = 4.11; bootstrap 95% *CI* [28.89, 32.16]), *t*_(__47__)_ = 1.44, *p* = 0.16. Nevertheless, in conducting the main analyses about the effect of gratitude exposure on felt/expressed gratitude, we planned to control for GQ-6 scores to avoid any potential statistical artifacts due to any potential difference in participants’ trait gratitude.

Similarly, at a descriptive level, preliminary analyses showed that participants in the two groups reported analogous habits and preferences on social media. In particular, at the onset of the study, both groups most frequently indicated Instagram as the favorite social networking site (40.00% in the control group; 50.00% in the gratitude group) and declared using groups on Facebook not regularly (*M* = 2.96, *SD* = 1.81 in the control group; *M* = 2.05, *SD* = 1.40 in the gratitude group).

Despite that Facebook was not the favorite social media of most of our participants, we observed a regular use of the groups during the manipulation implementation, with members visiting the groups almost daily. Exploratory analyses conducted on data coming from the Facebook posts revealed that, in the gratitude group, accomplices and experimenters’ posts received an average of 27.12 views (*SD* = 3.14), 4.5 likes/reactions (*SD* = 2.08), and 1.54 comments (*SD* = 1.63) per post. In the control group, the same posts received an average of 25.88 views (*SD* = 2.39), 5.35 likes/reactions (*SD* = 2.45), and 2.62 comments (*SD* = 2.83) per post. All types of social interactions induced by the Facebook posts did not statistically differ among groups, and none of the posted content ever reached less than 21 views. All posts received at least one like. Thus, although at the onset of the study they declared they were not used to visiting and/or using Facebook every day, these results suggest that participants actually visited our groups on a daily basis, showing acceptable levels of engagement in both groups.

### Effects of Gratitude Exposure on Feelings of Gratitude

To test the hypothesis that exposure to others’ grateful interactions on Facebook could improve feelings of gratitude, we ran a one-factorial (*Gratitude exposure on Facebook*: control vs. gratitude group) analysis of covariance (ANCOVA) controlling for participants’ trait gratitude (i.e., GQ-6 score) with the total scale of felt gratitude as the dependent variable. Despite our expectations, we found no significant difference in felt gratitude between the control group (*M* = 2.69, *SD* = 0.15; bootstrap 95% *CI* with 5,000 resamples [2.38, 3.01]) and the gratitude group (*M* = 2.70, *SD* = 0.16; bootstrap 95% *CI* [2.40, 3.01]), after controlling for trait gratitude score, *F*_(__1_, _46_) = 0.003, *p* = 0.95, mean squared error (*MSE*) = 0.58, partial η^2^ < 0.001. Effects of gratitude exposure were not significant also when distinguishing among the subscale of direct feelings of gratitude (i.e., gratitude directly felt by participants), *F*_(__1, 46)_ = 0.002, *p* = 0.96, *MSE* = 0.70, and the subscale of indirect feelings of gratitude (i.e., gratitude felt because of a resonance reaction to others’ feelings), *F*_(__1, 46)_ = 0.98, *p* = 0.33, *MSE* = 0.91. In other words, our results on feelings of gratitude (i.e., a non-significant effect of gratitude exposure on felt gratitude) were consistent with all the different nuances of felt gratitude we measured and considered as a dependent variable.

### Effects of Gratitude Exposure on Expression of Gratitude

We then proceeded with testing whether gratitude exposure on Facebook could augment gratitude expression through various channels (i.e., in person, via telephone calls, via instant messages, and via social media public posts). As expected, a one-factorial ANCOVA revealed that participants in the gratitude condition reported having expressed gratitude in person more frequently (*M* = 3.62, *SD* = 0.88; bootstrap 95% *CI* [3.29, 3.96]) than did participants in the control condition (*M* = 3.08, *SD* = 1.08; bootstrap 95% *CI* [2.64, 3.48]), after controlling for trait gratitude (GQ-6 score), *F*_(__1, 46)_ = 8.80, *p* = 0.005, *MSE* = 0.743, partial η^2^ = 0.161 (see Table 1). With respect to other channels to express gratitude, however, no other effects of the manipulation on expressed gratitude reached conventional levels of Fisherian statistical significance, *F*s_(__1, 46)_ < 2.91, *p*s > 0.09, partial η^2^s < 0.060.

**TABLE 1:** Average expression of gratitude in person and average scores obtained in the Satisfaction With Life Scale (SWLS) as a function of gratitude exposure on Facebook (control vs. gratitude exposure) and time (pre- vs. post-manipulation).

	**Gratitude exposure on Facebook**	
	**Control**	**Gratitude exposure**
	**Expression of gratitude in person**	
**Post-manipulation**		
*M (SD)*	3.08 (1.08)	3.62 (0.88)
*SEs*	0.22	0.18
*Bootstrap 95% CIs*	[2.64, 3.48]	[3.29, 3.96]
	**Satisfaction with life (SWLS)**	
**Pre-manipulation**		
*M (SD)*	4.70 (0.75)	4.53 (1.11)
*SEs*	0.15	0.23
*Bootstrap 95% CIs*	[4.42, 4.98]	[4.09, 4.96]
**Post-manipulation**		
*M (SD)*	4.62 (0.77)	4.81 (1.07)
*SEs*	0.15	0.22
*Bootstrap 95% CIs*	[4.33, 4.92]	[4.39, 5.23]

*Average expression of gratitude in person ranged from 1 “never” to 5 “very frequently,” while SWLS scores from 1 to 7, with higher scores indicating higher levels of satisfaction with life. Standard deviations (SDs) are displayed in parentheses. Bootstrap estimates for 95% confidence intervals (CIs) of the means were obtained with 5,000 resamples.*

### Effects of Gratitude Exposure on Well-Being

**FIGURE 2:**
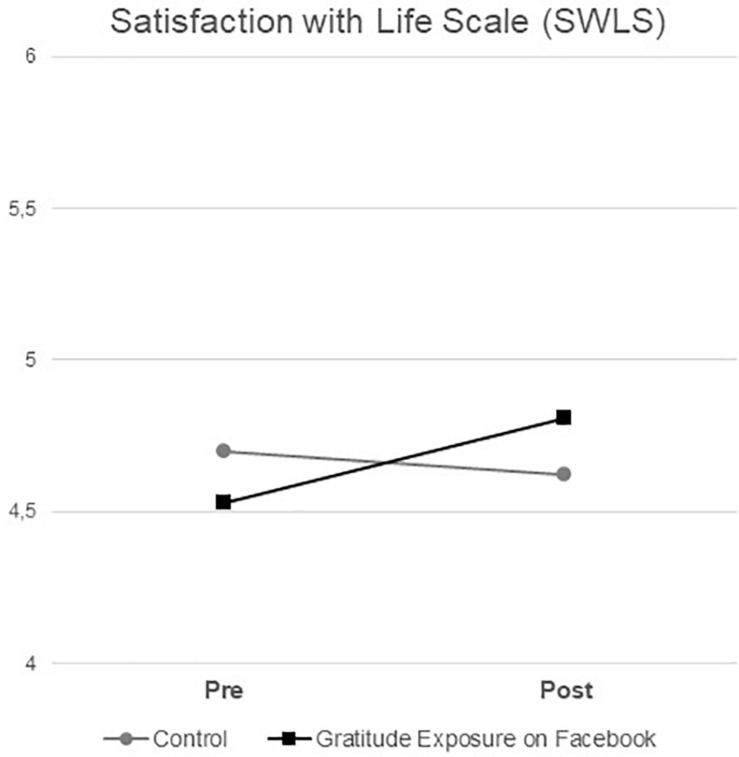
Satisfaction with life scale (SWLS) scores as a function of gratitude exposure on Facebook (control vs. gratitude exposure) and time (pre- vs. post-manipulation). SWLS scores ranged from 1 to 7, with higher scores indicating higher levels of satisfaction with life.

To test the hypothesis that gratitude exposure on Facebook could improve also well-being, we first ran a 2 (*Gratitude exposure on Facebook*: control vs. gratitude group) × 2 (*Time*: pre- vs. post-manipulation) repeated-measures ANOVA with participants’ satisfaction with life (i.e., SWLS score) as the dependent variable. Results showed a significant interaction effect of gratitude exposure and time on SWLS scores, *F*_(__1, 47)_ = 4.55, *p* = 0.04, *MSE* = 0.17, partial η^2^ < 0.088 (see Figure 2), demonstrating that observing others’ grateful interactions on Facebook actually ameliorated life satisfaction over the time. More specifically, after the manipulation, satisfaction with life augmented *only* in those participants assigned to the gratitude condition (*M* = 4.53, *SD* = 1.11, bootstrap 95% *CI* with 5,000 resamples [4.09, 4.96] before the manipulation; *M* = 4.81, *SD* = 1.07, bootstrap 95% *CI* [4.39, 5.23] after the manipulation), *t*_(__23__)_ = 2.15, *p* = 0.04, *dz* = 0.44, while it remained constant in participants assigned to the control condition (*M* = 4.70, *SD* = 0.75, bootstrap 95% *CI* [4.42, 4.98] before the manipulation; *M* = 4.62, *SD* = 0.77, bootstrap 95% *CI* [4.33, 4.92] after the manipulation), *t*_(__24)_ = 0.70, *p* = 0.42, *dz* = 0.16 (see Table 1). No main effects were significant, *F*s_(1, 47)_ < 1.61, *p*s > 0.21, partial η^2^s < 0.033.

Finally, we ran two distinct 2 (*Gratitude exposure on Facebook*: control vs. gratitude group) × 2 (*Time*: pre- vs. post-manipulation) repeated-measures ANOVAs with participants’ Positive and Negative Affect scores (i.e., PANAS subscales scores) as the dependent variables. Results revealed no significant interaction effect of gratitude exposure and time on Positive Affect scores, *F*_(__1, 47)_ = 0.13, *p* = 0.72, *MSE* = 0.14, partial η^2^ < 0.003. Specifically, Positive Affect scores did not vary over time, neither in participants assigned to the gratitude condition (*M* = 3.44, *SD* = 0.63, bootstrap 95% *CI* [3.19, 3.68] before the manipulation; *M* = 3.47, *SD* = 0.74, bootstrap 95% *CI* [3.17, 3.76] after the manipulation), *t*_(__23)_ = 0.33, *p* = 0.74, *dz* = 0.06, nor in those assigned to the control condition (*M* = 3.23, *SD* = 0.60, bootstrap 95% *CI* [3.01, 3.48] before the manipulation; *M* = 3.32, *SD* = 0.65, bootstrap 95% *CI* [3.07, 3.56] after the manipulation), *t*_(__24)_ = 0.79, *p* = 0.44, *dz* = 16. Analyses on participants’ negative affect revealed similar results, as the interaction effect of gratitude exposure and time on Negative Affect scores was not significant, *F*_(__1, 47)_ = 1.00, *p* = 0.32, *MSE* = 0.18, partial η^2^ < 0.021. Specifically, Negative Affect scores did not vary over time, neither in the gratitude condition (*M* = 2.22, *SD* = 0.52, bootstrap 95% *CI* [1.99, 2.40] before the manipulation; *M* = 2.19, *SD* = 0.52, bootstrap 95% *CI* [1.99, 2.40] after the manipulation), *t*_(__23)_ = 0.29, *p* = 0.77, *dz* = 0.08, nor in the control condition (*M* = 2.30, *SD* = 0.66, bootstrap 95% *CI* [2.06, 2.56] before the manipulation; *M* = 2.45, *SD* = 0.59, bootstrap 95% *CI* [2.23, 2.68] after the manipulation), *t*_(__24)_ = 1.00, *p* = 0.33, *dz* = 0.20. Lastly, with respect to both ANOVAs, no main effects were significant, *F*s_(__1, 47)_ < 1.43, *p*s > 0.24, partial η^2^s < 0.030. Table 1 summarizes the most relevant results of the current study.

## Discussion

Drawing on the theory of social learning (Bandura, 1968, 1977) and the most direct implications of emotional contagion (Hatfield et al., 1994), our pilot experiment aimed to test whether the exposure to others’ grateful interactions on Facebook enhanced participants’ feelings of gratitude (first hypothesis), their expression of gratitude (second hypothesis), and their general well-being (third hypothesis). Results partially supported our hypotheses. Specifically, we found that observing some accomplices’ gratitude exchange of posts and comments on Facebook actually *influenced* participants’ in-person expression of gratitude (i.e., gratitude expression in face-to-face interactions), supporting the hypothesized social learning effect (second hypothesis). By contrast, we failed to document a significant effect of gratitude exposure on their direct and subjective experiences of gratitude, i.e., the expected emotional contagion effect (first hypothesis). In other words, participants in the gratitude group did not seem to feel more grateful if compared with those in the control group. However, it is important to note that in the present research, we only evaluated participants’ expression of gratitude using self-report measures. Future studies are encouraged to make use of behavioral measures, directly observing and recording participants’ behaviors. With respect to the third hypothesis, exposure to others’ grateful messages on Facebook clearly *augmented* one component of subjective well-being, i.e., participants’ satisfaction with life. On the other hand, however, our results did not show any significant effect of gratitude passive observation on negative and positive affect.

With respect to gratitude learning and expression, our findings bring preliminary support for the theory of social learning (Bandura, 1968, 1977). Driven by the theory, we predicted and found that an exposure to others’ grateful interactions on Facebook was sufficient to make users express more gratitude themselves in face-to-face social circumstances. To our knowledge, this is the first experimental evidence in favor of a causal effect of observational learning in gratitude diffusion. Observing grateful behaviors of others may be enough to spontaneously learn from those behaviors and express more gratitude. Our findings also add to the pioneering experiments of Algoe et al. (2020), in which the authors demonstrated that observing a grateful social interaction makes an observer more helpful and affiliative toward both interaction’s actors. In this sense, our study complements such “witnessing effect” showing how that observer could be not only driven to experience more affiliative feelings but also influenced in her/his own tendency to express gratitude.

Notably, the present research is also the first attempt to explore the specific role that social media may play in the diffusion of grateful behaviors and, potentially, also other social attitudes (e.g., politeness and tendency to forgive). We purposely focused on a specific feature of social media—i.e., the possibility to observe and absorb from other users’ interactions by just staying in the field, without necessarily engaging in active communications or any other social effort—but many other social sites’ affordances could be of interests in this sense (e.g., the direct communication channel offered by Instagram’s Stories). Forthcoming, research will surely enlighten on this.

To better interpret our results, some critical issues should be considered. First of all, given the underpowered nature of our pilot study, further experiments—with a larger sample size—are absolutely required to replicate and extend the present findings. In other words, for the reported effects to be convincing and generalizable, they should be replicated by future research adopting either similar or alternative manipulations of gratitude exposure. In this respect, we highly suggest researchers interested in studying the effects of gratitude exposure on social media start from our preliminary results and respective effect sizes to estimate the *minimum* sample size required in future experiments (see also Brysbaert, 2019, for an adequate estimation of sample sizes in properly powered experimental tests).

Another critical point regards one of our main hypotheses. We postulated that observing others’ grateful posts and comments on Facebook might lead users to experience more gratitude as a result of emotional contagion (Kramer, 2014). However, participants exposed to others’ examples of gratitude did not report an augmented affective experience. To be precise, they did not feel a direct increase of grateful feelings, neither of positive affect. The only dimension that responded to gratitude exposure seem to be one component of well-being, but also the most cognition-mediated one, i.e., participants’ reported satisfaction with life. This result could be attributed to a lack of strength ties among our study’s participants (cf. Gilbert, 2012). According to Gilbert (2012), indeed, people share emotions within intimate relationships and with strong ties (usually close friends or family members), while they do not feel emotionally close to weak ties or persons less familiar to them, such as acquaintances (Preston and de Waal, 2002). Accordingly, Lin and Utz (2015) demonstrated that the emotional reaction to a social media post depends not only on the content of the post but also on the relationship between the poster and reader: The closer the relationship, the stronger will be the emotion induced by the post. In our study, participants did not know each other or were mere acquaintances. Thus, reading strangers’ posts expressing gratitude may have not triggered a proper process of emotional contagion.

Furthermore, the effect of exposure to strangers’ positive posts on affective response may also depend on the viewer’s characteristics. Specifically, it seems that individuals’ tendency to engage in social comparison influences the emotional effects of exposure to social media content (Valkenburg and Peter, 2013; Vogel et al., 2015). This means that a positive post from a stranger on Facebook can influence the affective response of viewers either in a positive or negative way, depending on whether they usually respond with social comparison or emotional contagion. In fact, De Vries et al. (2018) recently found that viewing strangers’ positive Instagram posts decreased positive affect among individuals with high social comparison orientation but increased positive affect among individuals with low social comparison orientation. Since we did not include an assessment of social comparison orientation in our procedure, these differences may have flattened the current results. Future studies are then encouraged to consider these and other individual differences to clarify effects of exposure to emotional expression through social media on users’ emotional responses.

Although we did not find a pure emotional contagion effect (i.e., an influence of gratitude exposure on gratitude feelings and related emotional benefits), we observed an improvement of a cognitive dimension of subjective well-being: Participants exposed to gratitude on Facebook reported higher levels of satisfaction with life. A cognitive process of social appraisal could have mediated this interesting result. Social appraisal effect consists in a change of our interpretation or evaluation of what is happening that is caused by someone else’s emotion, such that we feel toward those events or facts more like the other person does (Lazarus, 1991; Parkinson, 2011). Accordingly, it is possible that our participants inferred some other emotions during observation of gratitude interactions, and not only grateful feelings. Social appraisal effect probably led them not only to remember positive feelings associated with posts contents (Alkozei et al., 2018) but also to share with authors of the grateful posts the satisfaction that should arise in both actors of a gratitude exchange. Thus, such potential social appraisal of grateful others, together with the perception of being surrounded by positive and polite people, may have positively influenced individuals’ evaluation of their own life satisfaction. Future research is encouraged to test and confirm this interpretation.

The present experiment also has practical implications. Our results should be of practical interest to anyone involved in education and/or communication as, for instance, teachers, professional communicators, social media managers, members of institutions, and organizations’ employees, but also parents and other family members interested in spreading positive attitudes like grateful expressions within their familiar ties. Considering and possibly implementing strategies that trigger learning as a result of social observation or, more specifically, as a consequence of exposure to models on social media could help educational practices and contribute to improve learning, engagement, group cohesion, and emotional learning among students and employees from different fields. This would be also coherent with the emergent approach of Positive Technology, an approach that aims at investigating how information and communications technology (ICT)-based applications and services could be used to improve well-being and foster positive growth of individuals, groups, and institutions (Villani et al., 2016; Gaggioli et al., 2019). The application of such strategies on social media would activate a virtuous cycle of spontaneous and reciprocal learning and, consequently, stimulate the diffusion of positive attitudes like gratitude. Such a virtuous cycle could ultimately contribute to ameliorate society’s well-being.

## Data Availability Statement

The raw data supporting the conclusions of this article will be made available by the authors, without undue reservation.

## Ethics Statement

The studies involving human participants were reviewed and approved by the Ethical Committee of the Catholic University of the Sacred Heart of Milan (CERPS). The patients/participants provided their written informed consent to participate in this study.

## Author Contributions

SS and DV developed the idea for this empirical research and carried out the data analysis and described its procedure and results. SS and AD prepared the materials applied in the empirical study and were responsible for the implementation of the survey and data collection. SS, DV, and AD were involved in all steps of the manuscript preparation, including the writing of the introduction, the theoretical references, and the discussion, as well as the preparation of the figures and the table. CR mentored and supervised the whole work in all its phases, from the development of early original ideas to the review of the final manuscript. All authors contributed to the article and approved the submitted version.

## Conflict of Interest

The authors declare that the research was conducted in the absence of any commercial or financial relationships that could be construed as a potential conflict of interest.
